# A decision support system for multi-target disease diagnosis: A bioinformatics approach

**DOI:** 10.1016/j.heliyon.2020.e03657

**Published:** 2020-03-29

**Authors:** Femi Emmanuel Ayo, Joseph Bamidele Awotunde, Roseline Oluwaseun Ogundokun, Sakinat Oluwabukonla Folorunso, Adebola Olayinka Adekunle

**Affiliations:** a Department of Physical and Computer Sciences, McPherson University, Seriki Sotayo, Ogun State, Nigeria; b Department of Computer Science, University of Ilorin, Ilorin, Kwara State, Nigeria; c Department of Computer Science, Landmark University, Omu Aran, Kwara State, Nigeria; d Department of Mathematical Sciences, Olabisi Onabanjo University, Ago Iwoye, Ogun State, Nigeria; e Department of Computer Science, Adeyemi College of Education, Ondo State, Nigeria

**Keywords:** Bioinformatics, Computer science, Malaria, Typhoid fever, Sequence alignment, Expert system

## Abstract

Malaria and typhoid fever are revered for their ability to individually or jointly cause high mortality rate. Both malaria and typhoid fever have similar symptoms and are famous for their co-existence in the human body, hence, causes problem of under-diagnosis when doctors tries to determine the exact disease out of the two diseases. This paper proposes a Bioinformatics Based Decision Support System (BBDSS) for malaria, typhoid and malaria typhoid diagnosis. The system is a hybrid of expert system and global alignment with constant penalty. The architecture of the proposed system takes input diagnosis sequence and benchmark diagnosis sequences through the browser, store these diagnosis sequences in the Knowledge base and set up the IF-THEN rules guiding the diagnosis decisions for malaria, typhoid and malaria typhoid respectively. The matching engine component of the system receives as input the input sequence and applies global alignment technique with constant penalty for the matching between the input sequence and the three benchmark sequences in turns. The global alignment technique with constant penalty applies its pre-defined process to generate optimal alignment and determine the disease condition of the patient through alignment scores comparison for the three benchmark diagnosis sequences. In order to evaluate the proposed system, ANOVA was used to compare the means of the three independent groups (malaria, typhoid and malaria typhoid) to determine whether there is statistical evidence that the associated values on the diagnosis variables means are significantly different. The ANOVA results indicated that the mean of the values on diagnosis variables is significantly different for at least one of the disease status groups. Similarly, multiple comparisons tests was further used to explicitly identify which means were different from one another. The multiple comparisons results showed that there is a statistically significant difference in the values on the diagnosis variables to diagnose the disease conditions between the groups of malaria and malaria typhoid. Conversely, there were no differences between the groups of malaria and typhoid fever as well as between the groups of typhoid fever and malaria typhoid. In order to show mean difference in the diagnosis scores between the orthodox and the proposed diagnosis system, t-test statistics was used. The results of the t-test statistics indicates that the mean values of diagnosis from the orthodox system differ from those of the proposed system. Finally, the evaluation of the proposed diagnosis system is most efficient at providing diagnosis for malaria and malaria typhoid at 97% accuracy.

## 1 Introduction

Malaria is a life threatening disease common in temperate climate zones including Sub-Saharan Africa, Asia and the Americas. A female Anopheles mosquitoes carrying plasmodium parasite in their salivary glands is the transmitter of malaria (Poolphol et al., 2017). The severity of malaria rest on the class of this plasmodium parasite. Malaria could be a product of many sources such as insect stings, blood transfusion through contaminated needles or unscreened blood (Abduah and Karunamoorthi, 2016). When an infected source infects a person, the plasmodium parasites is injected into the blood and down to the liver for its life cycle. After a complete life cycle of the parasite in lever, it then travels through the circulatory system and attack red blood cells (Jan et al., 2018; Sajjad et al., 2016). Symptoms of malaria are high fever, sweating, vomiting, shaking, headache, muscle and joints pain, usually noticeable within a few weeks after infection.

Typhoid on the other hand is a bacteria illness caused by the *Salmonella enterica serotype Typhi* and transmitted through a human carrier in the form of contaminated food and water (Qamar et al., 2018; Abatcha et al., 2019). The bacteria attack the intestine and temporarily stayed in the blood stream. The bacteria are then transported by white blood cells in the liver and bone marrow, where they regenerate and re-enter the blood stream. The maturity period of typhoid is basically a maximum of two weeks and the illness can take several weeks. The symptoms include headaches, diarrhea, high fever, poor appetite, and body pains.

Both malaria and typhoid fever have similar symptoms and are famous for their co-existence in the human body i.e. malaria and typhoid can combine in human as malaria typhoid causing severe complexity in diagnosis. Precisely speaking, the joint infection of malaria typhoid in a host causes problem of under-diagnosis when doctors tries to determine the exact disease out of the two diseases. Malaria and typhoid fever have been identified by scholars as killer diseases accounting for the periodic death of several millions of people worldwide. This high mortality rate can be traced to reasons such as poor medical diagnosis methods and lack of competent medical personnel.

Bioinformatics is an interdisciplinary field of science involving the use of information technology to solve problems inherent in biology and computer science (Edwards et al., 2009). Research in bioinformatics includes algorithms designed for storage, retrieval, and data analysis. Bioinformatics is a fast developing field of science combining biology, information engineering, computer science, mathematics and statistics to examine and understand biological phenomena. It has practical applications in specific areas such as molecular biology and medical disease diagnosis. Sequence Alignment is a form of bioinformatics that uses various algorithms to locate functional subsets in biological sequences (whether DNA or protein) (Rosenberg, 2009). Sequence alignments can also be deployed to non-biological phenomena such as in natural language, clustering and financial data.

An expert system is an area of Artificial Intelligence (AI) designed to learn the skills of a human-expert coded in the form of rules (Yadav and Pandey, 2015). An expert system has been identified as a vibrant tool for the identification of various diseases such as skin diseases (melanoma, impetigo, and eczema), kidney diseases, meningitis, cerebral palsy, migraine, cluster headache, stroke, epilepsy, multiple sclerosis, parkinson, alzheimer and huntington disease (Amarathunga et al., 2015; Singla et al., 2014). Recently, a lot of researches has been geared towards the use of expert system for medical disease diagnosis and this has transformed to the emergence of technologically inclined medical consultation. Therefore, expert system is regarded as a decision support system in combination with other techniques in the field of AI for diseases diagnosis based on known symptoms (Horvitz et al., 1988). The main objectives of this paper are (1) To improve on existing systems that can only diagnose one disease at a time (2) To design benchmark sequences of symptoms for malaria, typhoid fever and malaria typhoid and (3) To design a bioinformatics approach for the identification and prediction of malaria, typhoid fever and malaria typhoid simultaneously.

The rest of this paper is organized as follows: Section 2 presents related work. Materials and methods is presented in Section 3. The implementation procedure and discussion is presented in Section 4. System evaluation of the proposed approach is well highlighted in Section 5. Section 6 presents the conclusion and future work.

## 2 Related work

Computer inspired tool such as sequence alignment algorithms can be deployed in medical diagnosis systems to check death ratio and reduce the stress of waiting to see a medical doctor. Medical diagnosis system is an emerging technology in the field of AI used to help health care experts in making efficient and appropriate clinical decisions (Shortliffe, 1987). Medical diagnosis systems in combination with bioinformatics inspired techniques can provide useful information on medical data under the knowledge supervision of a human expert. This useful information can assist medical experts in identifying disease categories in patients and provide timely intervention in the form of treatments advice (Wan and Fadzilah, 2006).

Researchers have developed several intelligent approaches for medical diagnosis systems in an attempt to identify disease category, reduce waiting time of patients, reduce health care service costs and increase service rate of medical experts. As seen in most studies (Bourlas et al., 1999; Alexopoulos et al., 1999; Ruseckaite, 1999; Manickam and Abidi, 1999; Zelic et al., 1999), intelligent approaches developed to assist experts in timely detection and prevention of diseases can only deal with one disease condition. Hence, it is important to develop a multi-target disease diagnosis system for the identification of two or more disease conditions in patients.

Oguntimilehin et al. (2013) presented a machine learning approach for clinical diagnosis of typhoid fever. The authors collected labelled dataset with severity levels of typhoid fever from medical experts. The labelled dataset comprises of diagnosis variables and severity levels of very low, low, moderate, high and very high as classes to create reasonable guidelines for the diagnosis of typhoid fever with 96% detection accuracy. The authors asserted that the system could lead to reduction in mortality rate and patient waiting time respectively. One limitation of their work was the problem of rule extraction, which, if overcome, could lead to better diagnosis accuracy. Samuel and Omisore (2013) proposed a mixture of fuzzy logic and neural network for the efficient diagnosis of typhoid fever. The mixture model provide a method that allows the neural network module to automatically optimize the diagnosis of typhoid fever by generating the diagnosis rules for the fuzzy inference system. The mixture model was reported to offer reliable diagnosis that is time efficient and less expensive. However, the proposed mixture model could lead to computational overhead due to unproven concept of weight adjustment in neural networks. Fatumo et al. (2013) designed a robust computer simulated medical expert based on input diagnosis variables as rules stored in the inference engine for the identification of different types of malaria and typhoid problems. The designed medical expert system offers effectiveness in use and accessibility, although insufficient rules and symptoms in the knowledge base can reduce the effectiveness of their designed system. Djam et al. (2011) designed a fuzzy expert system for the diagnosis and treatment of malaria based on degree of participation of each diagnosis variables using the root sum square and centre of gravity for reasoning and diagnosis decision respectively. The designed fuzzy expert system was able to provide reasonable diagnosis for malaria with some degree of confidence. The authors considered the designed fuzzy expert system to be user friendly and a means to ease medical consultations. The disadvantage of the system is centered on the problem of knowledge representation inherent to most rule based systems. Adehor and Burrell (2008) designed a simple differential diagnostic model for detecting malaria, typhoid and unknown-fever in the subregions of Africa based on signs and symptoms provided from interaction with the users. The designed model provides a more simplified way for entering signs and symptoms by taking responses from both a supervising user and the patient. This way of information entry reduces erroneous information and enhances the diagnosis accuracy. The designed differential diagnostic model could lead to delays, risks and expensive inefficient diagnosis due to multiple alternative solutions that may be similar. Aminu et al. (2016) proposed a predictive symptoms-based system rooted in the binary classification of Support Vector Machines (SVM) to enhanced joint classification of malaria and typhoid fever. The authors reported that the proposed predictive symptoms-based system represents a reliable substitute for disease diagnosis and the evaluation results indicates a low classification accuracy.

Samuel et al. (2013) proposed a Web-Based Decision Support System (WBDSS) rooted in Fuzzy Logic (FL) for the diagnosis of typhoid fever. The FL system is composed of a fuzzifier, fuzzy inference engine, and a defuzzifier for rules formulation, reasoning and diagnosis decision respectively. The results obtained showed that the proposed system is suitable for diagnosis problems. However, the fuzzy sets of fuzzy logic models cannot automatically adjust its linguistic variables to suit unseen conditions. Boruah and Kakoty (2019) provide a comparative analysis of different data mining techniques for the prediction and diagnosis of malaria. The study inferred that ensemble data mining techniques could be more efficient in the prediction and diagnosis of malaria than a single predictive model. The authors recognized that most literatures on disease diagnosis systems failed to test their systems on detection accuracy, simplicity and accessibility. Uzoka et al. (2011) proposed a combination of fuzzy logic and the Analytical Hierarchy Process (AHP) methods in the medical diagnosis of malaria. The fuzzy logic provides the rules needed to combine the multiple diagnosis decision variables supported by AHP in order to determine the relative importance of each variable in the diagnostic decision making process. The results of the research proved effective for non-expert medical practitioner in the diagnosis of malaria. The limitation of the system hinges on the problem of knowledge representation identified with fuzzy logic systems. Mutawa and Alzuwawi (2019) presented a multi-layered rule-based expert system for detecting uveitis. The rules combination on the diagnosis variables decreases as the network propagate from the input layers to the output layer. The network design assist in deciding the primary signs and symptoms of some diseases needed to evaluate the probability of that disease instead of integrating all the disease diagnosis variables. The system represents an intelligent guideline for young medical doctors in providing accurate treatment advice to patients. The system provides easy adaptability to unseen conditions through its unique multilayer design. Conversely, the system has no technique that can mitigate input errors of signs and symptoms from users.

### 2.1 Bioinformatics

**Figure 1:**
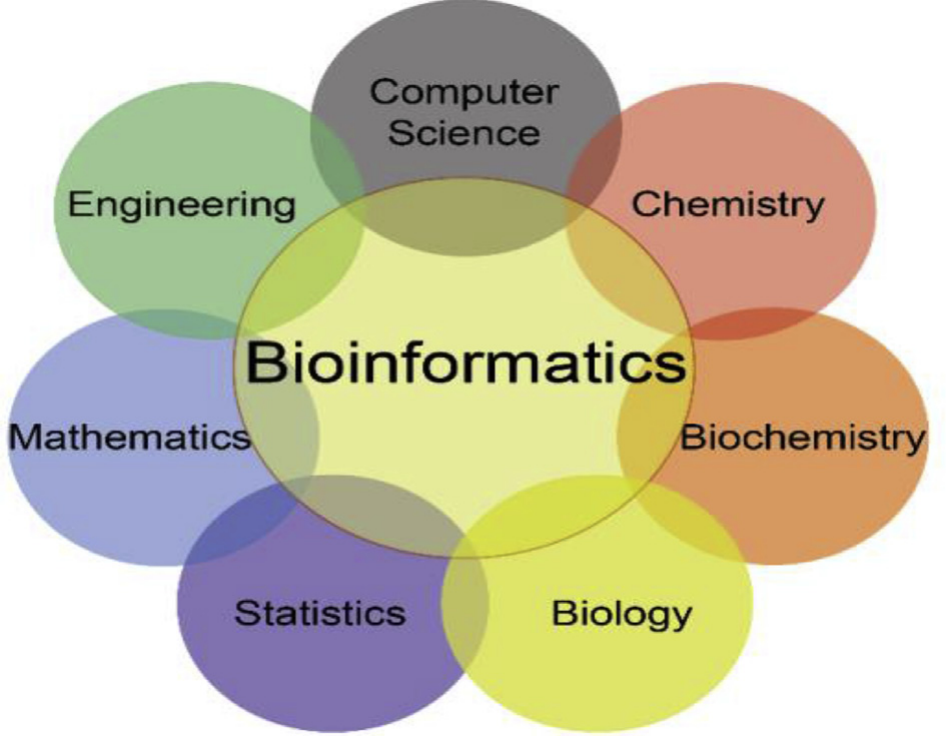
Bioinformatics disciplines (**Source**: Diniz and Canduri, 2017).

Bioinformatics is an interdisciplinary field of science consisting of tools developed to gain knowledge about biological phenomena (Edwards et al., 2009). It is a technology initially designed for the practical purpose of introducing pattern into the big data generated by the modern development in molecular biology. The bioinformatics technology started with the idea of developing computer inspired tools for locating functional patterns in biological sequences e.g. locations of functional structures in Deoxyribonucleic Acid (DNA). Bioinformatics is a fast developing field of science combining biology, information engineering, computer science, mathematics, chemistry and statistics to derive useful knowledge from biological phenomena (See Figure 1). One common area of its application include medical disease diagnosis.

### 2.2 Sequence alignment methods

Sequence Alignment is a form of bioinformatics tool designed for the comparison of two or more sequences in order to derive important biological knowledge (Behbahani et al., 2016). It is used to discover both patterns and functional connection between sequences. Alignment locates similarity grade between text sequences and pattern sequences. Most sequence alignment employs divide-and-conquer approach for optimal alignment scores.

The functional behaviour of an unknown pattern can be predicted by simply employing sequence alignment. The optimum similarity of the unknown pattern after alignment with a database of known text sequences is normally assumed as the functional information contained in the pattern. There are predominantly two techniques of sequence alignment: global alignment and local alignment.

#### 2.2.1 Global alignment

In global alignment comparison is done from start till finish of the pattern to locate the optimal alignment. This kind of alignment followed the Needleman-Wunsch algorithm (Needleman and Wunsch, 1970). This algorithm is very often used in many computer science applications. Global alignment method is most recommended for sequences that are identical in length.

#### 2.2.2 Local alignment

Sequences with no identical length can be matched with local alignment technique. It divides sequences into subsets and compare subsets of all possible lengths. This kind of alignment followed the Smith-Waterman algorithm (Smith and Waterman, 1981).

These two basic alignment techniques are known to follow the popular divide-and-conquer approach.

### 2.3 Gap penalty

A Gap penalty is a technique derived to allow for more character matching between closely related sequences. When comparing sequences, the use of gaps in the sequences can allow more characters to be matched by an alignment algorithm than it's possible in normal alignment. However, to arrive at suitable alignment it is essential to control the length and number of gaps in an alignment. The three basic types of gap penalties are constant, linear and affine (Manikandan and Ramyachitra, 2017).

#### 2.3.1 Constant

**Figure 2:**
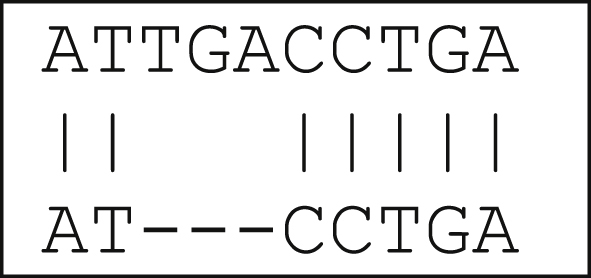
1-gap alignment.

This is the most basic type of gap penalty where a fixed negative score is assigned to every gap, irrespective of its length. For example, aligning two sequences as in Figure 2, with '-' showing a 1-gap alignment. Assume a 1 is assigned for every match and -1 for every gap, then total score is 7 − 1 = 6 as computed by (1).

(1) Score=numberofmatches−numberofgaps

#### 2.3.2 Linear

The linear gap penalty in contrast to constant gap penalty consider the length (L) of each insertion/deletion in the gap. Hence, if the penalty for each gap is Y and the length of the gap is L; the resultant gap penalty is the product of the two YL. This technique discourages lengthy gaps, with total score decreasing for each additional gap. For example, the total score for Figure 2 using linear gap penalty is 7 − 3 = 4 as computed by (2).

(2) Score=numberofmatches−(Y·L)

#### 2.3.3 Affine

This is a blend of the constant and linear gap penalty. It is the most common of the gap penalty types. The affine gap penalty is of the form.

(3) Score=numberofmatches−(X+Y·L)

where X is the gap opening penalty, Y the gap extension penalty and L the length of the gap. Gap opening denotes the cost necessary to open a gap of any length, and gap extension is the cost for each additional length to an existing gap. Although, the value of X and Y varies according to purpose and thus the values cannot be ascertained. If the purpose is to find closely related matches, a bigger gap penalty is needed to discourage gap openings. Conversely, if the purpose is simply to find a less closely related match, then a reduced gap penalty is recommended.

### 2.4 Expert systems

The uncultivated habit of people to visit the hospital for regular check-ups coupled with their busy schedule, has triggered the emergence of medical diagnosis systems as an alternative for human experts. The wide acceptance of these medical diagnosis systems has translated to an alteration from human consultation to system consultation. Medical diagnosis system is an expert system with coded knowledge of some domain experts which can categorize diseases based on selected symptoms (Lingiardi et al., 2015; Weiss et al., 1978; Moses, 2015; Mutawa and Alzuwawi, 2019). These coded knowledge based on some inference mechanisms are deployed by the system for making smart decisions.

Moreso, the deployment of these medical diagnosis systems can greatly assist medical staff in the discharge of quality health care services. AI as an umbrella word in intelligent computing, is the design and implementation of machines behaving at the level of a human expert. Expert systems have carved out a niche for itself in AI compared to other machine learning techniques. The most recognised area of expert systems application is inherent in the health care domain for detection and prevention of diseases (Pai et al., 2006). The friendly user interface and explanation facilities of expert systems has made them the most popular tool for problem solving.

## 3 Materials and methods

**Figure 3:**
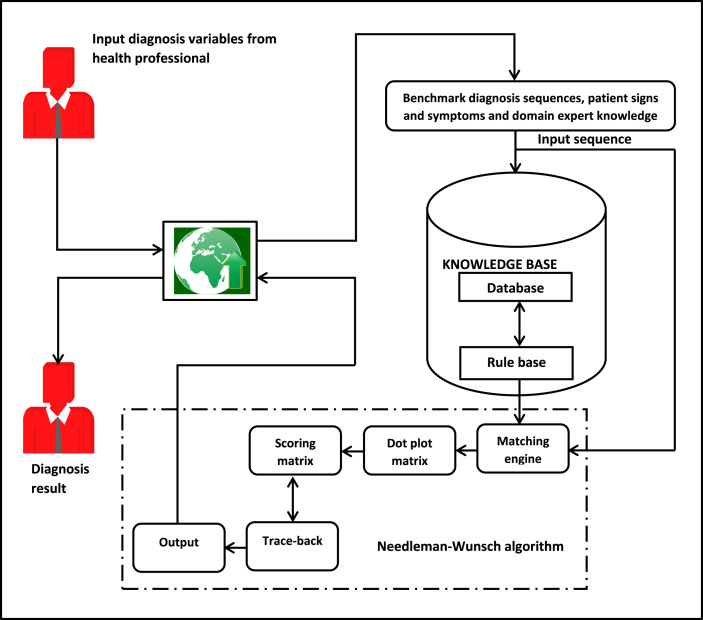
Architecture of BBDSS for multi-target disease diagnosis.

The architecture of the proposed Bioinformatics Based Decision Support System (BBDSS) for multi-target disease diagnosis is presented in Figure 3.

**Figure 4:**
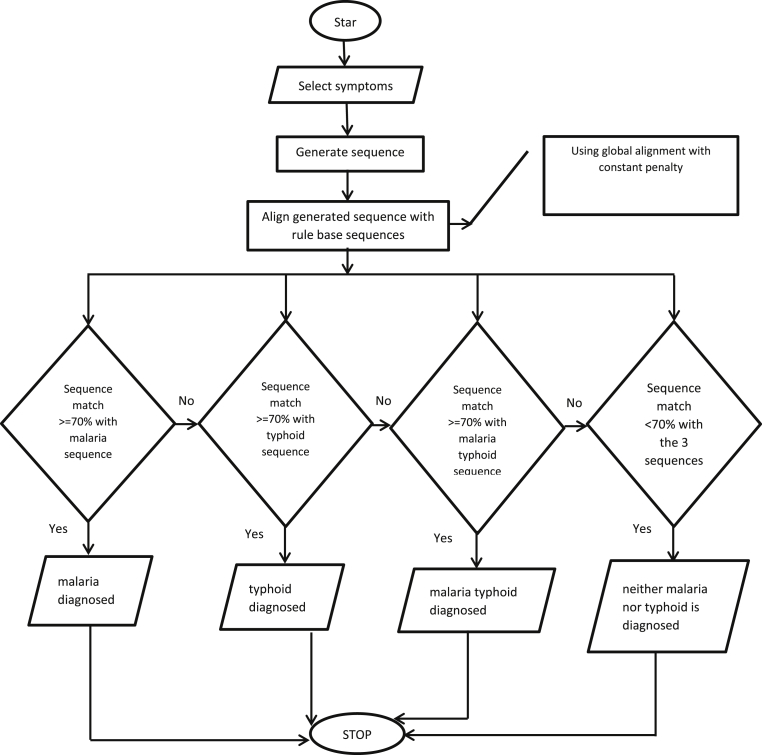
Flowchart of the proposed system.

Figure 3 consists of a fusion of expert system and sequence alignment techniques for the diagnosis of Malaria Fever (MF), Typhoid Fever (TF) and Malaria Typhoid Fever (MTF). The architecture takes input diagnosis variables through the browser representing benchmark diagnosis sequences for the three disease conditions, patient signs and symptoms and domain expert knowledge. The browser is the interface through which the users (health professional and domain expert) interact with the system and provide diagnosis results to the outside world. The Knowledge base comprises of the database and Rule Base (RB). The database stores benchmark diagnosis sequences, patient signs and symptoms and domain expert knowledge while the RB is made up of a set of IF-THEN rules depicting the benchmark diagnosis variables for each disease conditions of MF, TF and MTF respectively. The sequence alignment component receives as input the patient signs and symptoms (input sequence) supplied by the patient and applies global alignment technique with constant penalty for the matching between the input sequence and the three benchmark sequences in turns. The global alignment technique with constant penalty applies its pre-defined process to generate optimal alignment and determine the disease condition of the patient through comparing the alignment scores for the three benchmark diagnosis sequences. Finally, the best optimal alignment score is returned as the diagnosis result for the patient. The full details of the sequence alignment component showing the process flow is also presented in Figure 4.

### 3.1 Diagnosis variables

Given an input sequence X of patient signs and symptoms and benchmark sequences Y of domain expert rules for the diagnosis of MF, TF and MTF respectively. Let Eqs. (4) and (5) represent input and benchmark diagnosis sequences respectively;

(4) $$X=x_{1}x_{2}\ldots x_{n}$$

(5) $$Y=y_{1}y_{2}\ldots y_{m}$$

Such that $x_{i}$ and $y_{i}$ represents joint disease diagnosis variables for the possible diagnosis of MF, TF and MTF as in Table 1. Table 1 shows the joint disease diagnosis variable, value and abbreviation code. Depending on the input sequence per patient in alignment with the benchmark diagnosis sequences, MF, TF or MTF can be diagnosed.

**Table 1:** MF, TF, MTF diagnosis variables.

SN	MF, TF, MTF diagnosis variable	Value	CODE
1	Generalized Body Pain	Y/N	GBP
2	Generalized Body Discomfort (Malaise)	Y/N	GBD
3	Generalized Body Weakness	Y/N	GBW
4	Sweating Profusely	Y/N	SP
5	Vomiting	Y/N	VMT
6	Loss of Appetite	Y/N	LOA
7	Bitter taste in your Throat	Y/N	BT
8	Diarrhea (discharging faeces from the bowels frequently in liquid form)	Y/N	DHE
9	Type of Fever (Intermittent or Remittent)	I/R	TOF
10	Abdominal Distension (Swelling Stomach)	Y/N	AD
11	Abdominal Pain	Y,N	AP
12	Constipation	Y/N	CON
13	Loss of Weight	Y/N	LOW
14	Extreme Muscle Weakness	Y/N	EMW
15	Confusion	Y/N	CF
16	Irrational Talking	Y/N	IRR
17	Epistaxis (Bleeding nose)	Y/N	EPI

MF = Malaria Fever, TF = Typhoid Fever, MTF = Malaria Typhoid Fever, Y=Yes, N=No.

### 3.2 Database

The database stores patient interaction with the system providing symptoms details. The database is a repository storing the benchmark diagnosis sequences, patient signs and symptoms and domain expert knowledge. It receive input diagnosis variables and the domain expert knowledge through the browser interface. Hence, the authors sought and obtained ethical approval from the Landmark University Research Ethical Board. This ethical approval was given by the Landmark University in collaboration with its medical center.

### 3.3 Rule base

The rule base for MF, TF and MTF is composed of the benchmark diagnosis variables combined by a set of IF-THEN rules in which the IF-parts consist of the diagnosis variables combined by the AND operator while the THEN-parts involve the diagnosis decisions. The rules that constitute the benchmark sequences were intelligently formulated with the knowledge of domain experts. Table 2 represents the rule base of benchmark sequences for the three disease conditions. The three disease conditions have joint diagnosis variables but different diagnosis values. When an input sequence is aligned with the benchmark sequences a rule is fired for a disease condition with the maximum alignment score, otherwise the diagnosis result returns no decision if all the alignment scores is below a specified percentage threshold.

**Table 2:** MF, TF, MTF benchmark sequences.

Disease	GBP	GBD	GBW	SP	VMT	LOA	BT	DHE	TOF	AD	AP	CON	LOW	EMW	CF	IRR	EPI
MF	Y	Y	Y	Y	Y	Y	Y	Y	I	N	N	N	N	N	N	N	N
TF	N	N	Y	N	Y	Y	N	Y	R	Y	Y	Y	Y	Y	Y	Y	Y
MTF	Y	Y	Y	Y	Y	Y	Y	Y	R	Y	Y	Y	Y	Y	Y	Y	Y

### 3.4 Dot plot matrix

A dot plot matrix or similarity matrix is constructed to permutate all the possible alignment between a given input diagnosis sequence and all the benchmark diagnosis sequences. In order to characterise all the possible combinations of variables and their resultant scores a similarity matrix is used. The similarity matrix is defined by an alignment matrix and scoring matrix.

### 3.5 Alignment

#### 3.5.1 Alignment operation

Given two sequences $X=x_{1}x_{2}\ldots x_{n}$ and $Y=y_{1}y_{2}\ldots y_{m}$ defined over an alphabet ∑. An alignment operation is a pair (x,y)∈(∑∪{−}≠(−,−)). Note that − ∉ ∑ but $\;x,y\in \sum^{⋆}$. We call (x, y).

• substitution iff x≠− and y≠−
• deletion (del) iff y = −
• insertion (in) iff x = −

#### 3.5.2 Alignment matrix

Let n=|X| and m=|Y|.Alignment matrix of X and Y is the (n+1)×(m+1)-matrix defined by (6):

(6) $$D_{ij}=D_{w}\left(x_{1\ldots i},y_{1\ldots j}\right)=min\left\{w\left(X,\;Y\right)\right\}$$

Given (X,Y) is alignment of $x_{1\ldots i},y_{1\ldots j}$, where w is the cost function.

### 3.6 Scoring matrix

We use a scoring scheme or cost function that simply give a value of 1 for each match, and 0 for mismatch using constant penalty as in (1). A simple scoring scheme (7) is used for the alignment between an input diagnosis sequence and benchmark diagnosis sequences, since no gap allowance is considered in the system. i.e., all sequences are of equal lengths.

(7) $$w\left(x,\;y\right)=\{\begin{array}{cc} 1 & iff\;x=y\\ 0 & iff\;x\neq y \end{array}$$

### 3.7 Trace-back

Once the alignment matrix with the cost function is computed, the entry D{nm} provides the maximum score among all possible alignments. To compute optimal alignment, you start from the bottom right cell as follows:

• Start in (n, m). For every (i, j) determine optimal case.
• Compare the value with the three possible sources (match, insert, and delete)
• Sequence of trace arrows with maximum trace gives optimal alignment.

Hence, the number of possible global alignments between an input diagnosis sequence and a benchmark diagnosis sequence of length N can be represented as in (8).

(8) $$n\left(x,y\right)=\frac{2^{2N}}{\sqrt{\pi N}}$$

### 3.8 Research hypotheses

In order to investigate if the mean score of the proposed system is statistically equal to the orthodox system, the following research hypotheses were formulated:

• H0: μ1 = μ2: The paired mean score between the orthodox system and the proposed system are equal, that is, differ on average by a small margin at most.
• H1: μ1 ≠ μ2: The paired mean score between the orthodox system and the proposed system are not equal, that is, differ on average by a large margin.

where H0 denotes the null hypothesis and H1 denotes the alternative hypothesis.

## 4 Implementation results and discussion

The proposed diagnosis system was implemented using a java programming language which runs on Netbeans IDE 8.0.2 environment and MySQL as the database management system.

**Figure 5:**
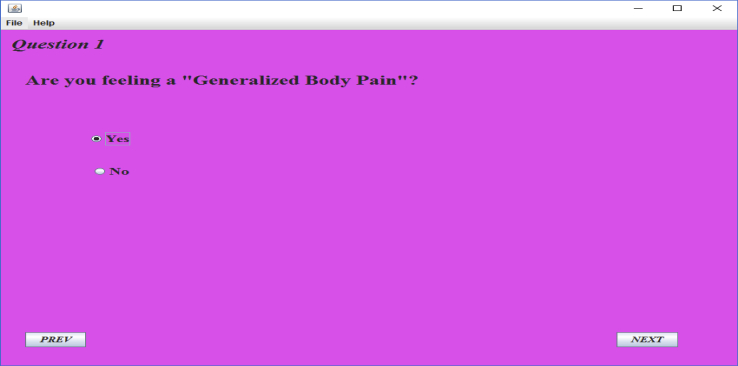
Patient diagnosis pane.

Figure 5 presents an instance interface through which a health professional enter the input diagnosis value in the form of signs and symptoms for a diagnosis decision. The health professional can enter the next input diagnosis value by pressing the next button. The diagnosis information were gathered from experts about symptoms of malaria and typhoid. These symptoms led to a total of 17 questions being asked on the GUI of the program with a “Yes” or “No” option. Any of the selected option generates a character (i.e. ‘Y’ for a “Yes” and ‘N’ for a “No”). These characters form a sequence of string at the end of the questions. Three benchmark sequences of strings are being stored in the rule base of the program, which represents malaria, typhoid and malaria typhoid sequences respectively.

**Figure 6:**
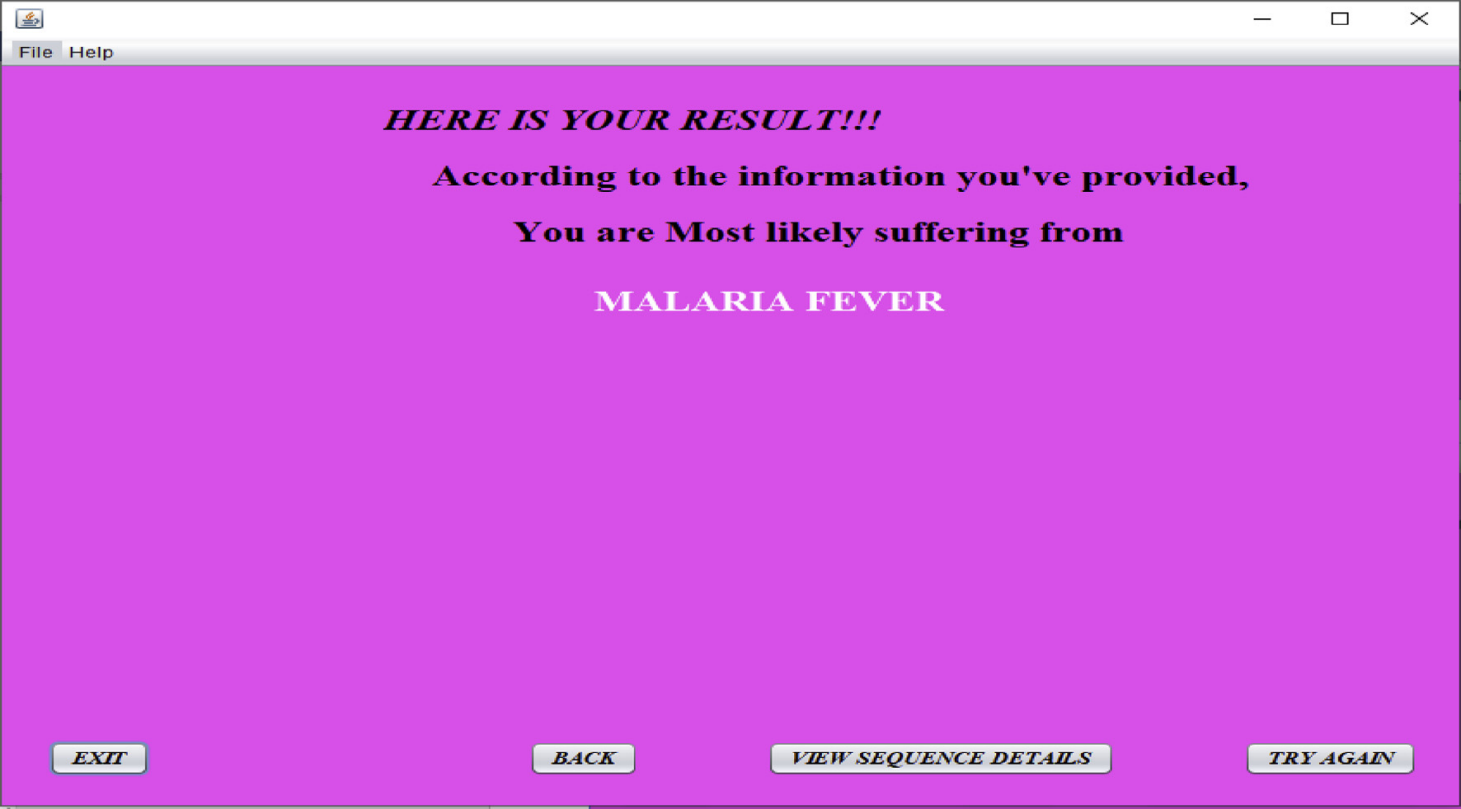
Diagnosis decision.

The string generated is then aligned with those in the rule base for comparison. The percentage of matches between the three sequences determines what disease the patient is most likely suffering from as in Figure 6. For example, if the generated string has a higher percentage of match with malaria sequence than that of the two other sequences, then the patient is most likely suffering from malaria. The proposed system is designed in such a way that if the percentage of matches between the generated sequence and the three benchmark sequences in the rule base is not up to 70%, then the patient is most likely not suffering from any of the three diseases. Hence, the patient is advised to visit the medical doctor (See Figure 4).

**Figure 7:**
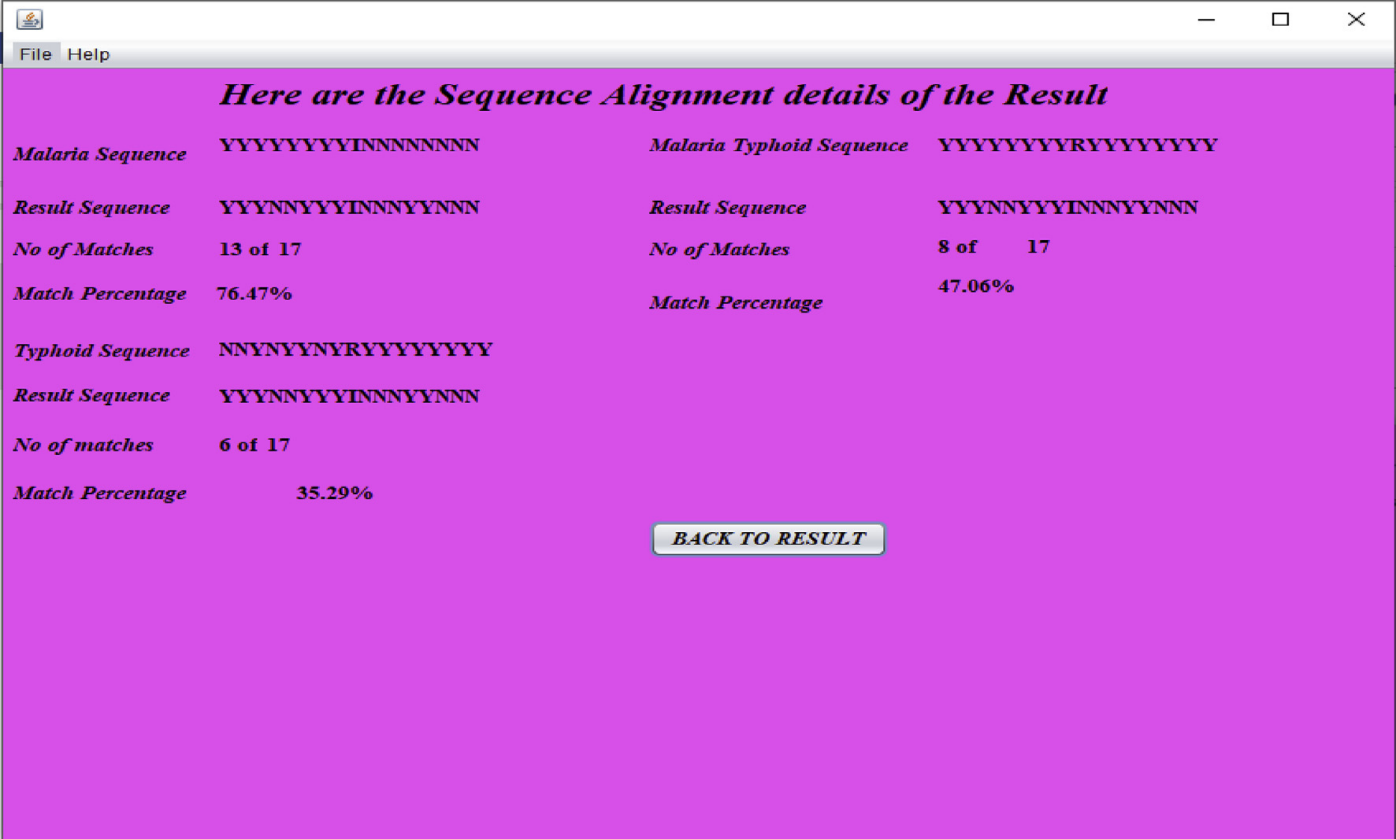
Sequence alignment result for patient with ID ‘‘001’’.

When the ‘‘View Sequence Details’’ button in Figure 6 is pressed, the result of sequence alignment details for a given patient is displayed as in Figure 7. This result represents the diagnosis results leading to a diagnosis decision for the patient with ID ‘‘001’’.

Table 3 represents the input diagnosis sequences of fifteen patients for MF, TF and MTF. Column 1 represent patient identification numbers. Columns 2–18 represents the diagnosis variables. Columns 19, 20 and 21 depicts the diagnosis scores for MF, TF and MTF respectively. The last column represents the diagnosis decision at each instance for the fifteen patients.

**Table 3:** Input diagnosis sequences for MF, TF and MTF.

ID	GBP	GBD	GBW	SP	VMT	LOA	BT	DHE	TOF	AD	AP	CON	LOW	EMW	CF	IRR	EPI	Score			Diagnosis
																		MF	TF	MTF	
001	1	1	1	0	0	1	1	1	I	0	0	0	1	1	0	0	0	0.7647	0.3529	0.4706	malaria
002	1	0	1	1	1	0	0	1	I	1	0	0	0	0	0	0	1	0.7059	0.4118	0.4118	malaria
003	0	0	1	0	0	1	0	1	R	0	1	0	1	1	0	1	1	0.3529	0.7647	0.5294	typhoid
004	1	1	1	0	1	0	0	1	I	1	1	1	1	0	1	0	1	0.4706	0.6471	0.6471	neither
005	0	1	1	0	1	1	0	0	R	1	1	1	1	1	1	1	1	0.2353	0.8824	0.7647	typhoid
006	0	0	0	1	0	0	1	0	R	0	0	1	0	0	1	1	0	0.4118	0.3529	0.3529	neither
007	1	1	0	1	1	1	1	0	I	0	1	0	0	1	0	0	1	0.7059	0.2941	0.5294	malaria
008	1	1	1	1	1	1	1	1	R	1	1	1	1	1	1	1	0	0.5294	0.7059	0.9412	malaria typhoid
009	0	0	1	0	1	1	0	0	R	0	1	1	1	0	1	1	1	0.2941	0.8235	0.5882	typhoid
010	1	1	1	1	1	0	1	1	R	1	1	1	1	1	0	1	1	0.4706	0.6471	0.8824	malaria typhoid
011	1	1	1	1	0	1	1	1	R	0	1	0	0	0	1	1	1	0.6471	0.4706	0.7059	malaria typhoid
012	0	1	0	1	1	1	1	0	I	0	0	0	0	0	0	0	0	0.8235	0.1765	0.2941	malaria
013	0	1	1	1	1	1	1	0	I	1	0	1	1	1	0	1	1	0.5294	0.5882	0.7059	malaria typhoid
014	0	0	1	1	1	1	0	1	R	1	1	0	1	1	1	1	1	0.3529	0.8824	0.7647	typhoid
015	0	0	0	0	0	1	0	0	R	1	1	1	1	0	0	1	1	0.1765	0.7059	0.4706	typhoid

1 = Yes, 0 = No, I = Intermittent, R = Remittent.

**Figure 8:**
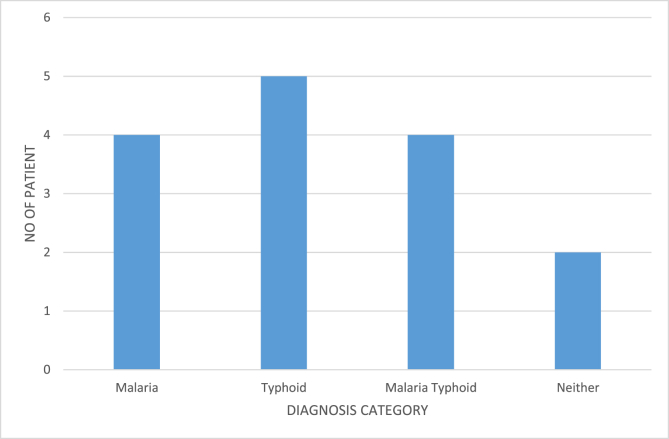
Diagnosis category Vs Number of patients.

Figure 8 presents the diagnosis category against the number of patients for the fifteen patients.

## 5 System evaluation

Since the diagnosis of malaria, typhoid and malaria typhoid is independent of one another but having joint diagnosis variables, a one-way ANOVA was used in order to compare the means of the three independent groups (malaria, typhoid and malaria typhoid) to determine whether there is statistical suggestion that the associated values on the diagnosis variables means are significantly different.

In the proposed system, the diagnosis variables is the health professional's inputs (Y/N) to diagnose a given disease condition, and disease status is an indicator about whether or not the patient have (0 = malaria, 1 = typhoid, 2 = malaria typhoid). We use ANOVA to test if there is a statistically significant difference in diagnosis variables with respect to disease status. Diagnosis variables will serve as the dependent variable, and disease status will act as the independent variable.

From Table 4, we conclude that the mean of the values on diagnosis variables is significantly different for at least one of the disease status groups (*F* (2, 351) = 9.194, *p* < 0.005). Since the ANOVA alone is considered insufficient to tell us explicitly which means were different from one another, multiple comparisons tests was further used.

**Table 4:** ANOVA.

	Sum of Squares	Df	Mean Square	F	Sig.
Between Groups	0.267	2	0.133	9.194	.000
Within Groups	5.093	351	0.015		
Total	5.359	353			

From the ANOVA results, we ascertained that there are statistically significant differences between the groups as a whole. From Table 5, multiple comparisons shows which groups differed from each other. The Tukey post hoc test was used due to its simplicity and preferred test for conducting post hoc tests on a one-way ANOVA. Table 5 shows that there is a statistically significant difference in the values on the diagnosis variables to diagnose the disease conditions between the groups of malaria and malaria typhoid (p = 0.000). However, there were no differences between the groups of malaria and typhoid fever (p = 0.141), as well as between the groups of typhoid fever and malaria typhoid (p = 0.520).

**Table 5:** Multiple Comparisons (measure mean difference based on the benchmark sequences).

(I) Category	(J) Category				95% Confidence Interval	
		Mean Difference (I-J)	Std. Error	Sig.	Lower Bound	Upper Bound
0	1 2	-.0422215 -.0707797∗	.0222496 .0173578	.141 .000	-.094590 -.111635	.010147 -.029925
1	0 2	.0422215 -.0285582	.0222496 .0261836	.141 .520	-.010147 -.090186	.094590 .033070
2	0 1	.0707797∗ .0285582	.0173578 .0261836	.000 .520	.029925 -.033070	.111635 .090186

∗ The mean difference is significant at the 0.05 level, 0 = malaria, 1 = typhoid, 2 = malaria typhoid.

In order to evaluate the performance of the proposed diagnosis system in terms of results accuracy, we performed a comparative analysis of one hundred and twenty random system diagnostic findings with those obtained from the orthodox method equivalent as shown in Table 6a, Table 6b, Table 6c. Table 6a, b, c gives the total diagnosis accuracy of the proposed system with accuracy of 38.1565, 38.703 and 39.5497 respectively. The mean accuracy can be computed by the summation of the three accuracies divided by the 120 data points.

**Table 6a:** Comparative analysis.

Patient ID	DROM	DRPS	$e_{i}$	(1$-\;e_{i}$)	Disease status
1	0.8128	0.7647	0.0481	0.9519	malaria
2	0.7638	0.7059	0.0579	0.9421	malaria
3	0.7925	0.7647	0.0278	0.9722	typhoid
4	0.8996	0.8824	0.0172	0.9828	malaria typhoid
5	0.9824	0.9412	0.0412	0.9588	typhoid
6	0.9412	0.8745	0.0667	0.9333	malaria
7	0.8235	0.7864	0.0371	0.9629	malaria
8	0.8824	0.8231	0.0593	0.9407	malaria typhoid
9	0.7059	0.6942	0.0117	0.9883	neither
10	0.8235	0.7864	0.0371	0.9629	malaria typhoid
11	0.7059	0.6942	0.0117	0.9883	neither
12	0.8824	0.8231	0.0593	0.9407	malaria
13	0.7059	0.6942	0.0117	0.9883	neither
14	0.8150	0.7833	0.0317	0.9683	typhoid
15	0.8525	0.7814	0.0711	0.9289	typhoid
16	0.8688	0.8134	0.0554	0.9446	malaria typhoid
17	0.8868	0.8113	0.0755	0.9245	malaria typhoid
18	0.7730	0.7025	0.0705	0.9295	malaria
19	0.8753	0.8227	0.0526	0.9474	typhoid
20	0.6731	0.6221	0.051	0.949	neither
21	0.7654	0.7243	0.0411	0.9589	malaria
22	0.9388	0.8267	0.1121	0.8879	typhoid
23	0.9134	0.8544	0.059	0.941	malaria
24	0.7674	0.7215	0.0459	0.9541	malaria
25	0.7445	0.7087	0.0358	0.9642	typhoid
26	0.8653	0.8262	0.0391	0.9609	malaria
27	0.8444	0.8045	0.0399	0.9601	malaria typhoid
28	0.8529	0.8153	0.0376	0.9624	typhoid
29	0.7806	0.7234	0.0572	0.9428	malaria
30	0.7441	0.7167	0.0274	0.9726	malaria
31	0.7402	0.7014	0.0388	0.9612	malaria
32	0.7961	0.7392	0.0569	0.9431	malaria typhoid
33	0.7964	0.7554	0.041	0.959	malaria
34	0.9581	0.9221	0.036	0.964	typhoid
35	0.8298	0.7552	0.0746	0.9254	malaria typhoid
36	0.7515	0.7261	0.0254	0.9746	malaria
37	0.8704	0.8278	0.0426	0.9574	typhoid
38	0.7041	0.6447	0.0594	0.9406	neither
39	0.6762	0.6255	0.0507	0.9493	neither
40	0.7719	0.7435	0.0284	0.9716	malaria
**Total**			**1.8435**	**38.1565**	

DROM = Diagnosis Results of the Orthodox Method, DRPS = Diagnosis Results of the Proposed.

System, $e_{i}$ = error in diagnosis, and (1$-\;e_{i}$) = accuracy of the proposed system.

**Table 6b:** Comparative analysis.

Patient ID	DROM	DRPS	$e_{i}$	(1$-\;e_{i}$)	Disease status
41	0.7560	0.7157	0.0403	0.9597	typhoid
42	0.8692	0.7841	0.0851	0.9149	malaria
43	0.8110	0.7981	0.0129	0.9871	malaria typhoid
44	0.8419	0.7898	0.0521	0.9479	malaria typhoid
45	0.8154	0.7628	0.0526	0.9474	malaria
46	0.8641	0.8124	0.0517	0.9483	typhoid
47	0.7765	0.7283	0.0482	0.9518	malaria
48	0.8477	0.7768	0.0709	0.9291	malaria
49	0.8585	0.8161	0.0424	0.9576	typhoid
50	0.8395	0.7827	0.0568	0.9432	malaria
51	0.7357	0.6742	0.0615	0.9385	neither
52	0.8660	0.8098	0.0562	0.9438	malaria
53	0.7165	0.6731	0.0434	0.9566	neither
54	0.8125	0.7815	0.031	0.969	malaria typhoid
55	0.8614	0.8198	0.0416	0.9584	malaria typhoid
56	0.8524	0.8354	0.017	0.983	malaria
57	0.7407	0.7297	0.011	0.989	malaria
58	0.8498	0.8192	0.0306	0.9694	malaria
59	0.7322	0.7157	0.0165	0.9835	typhoid
60	0.8082	0.7977	0.0105	0.9895	malaria typhoid
61	0.8734	0.8671	0.0063	0.9937	typhoid
62	0.7345	0.6924	0.0421	0.9579	neither
63	0.8548	0.8486	0.0062	0.9938	malaria typhoid
64	0.8101	0.7948	0.0153	0.9847	malaria
65	0.8277	0.8042	0.0235	0.9765	malaria
66	0.8415	0.8171	0.0244	0.9756	typhoid
67	0.8613	0.8537	0.0076	0.9924	malaria
68	0.7109	0.6898	0.0211	0.9789	neither
69	0.7645	0.7571	0.0074	0.9926	malaria
70	0.8051	0.7732	0.0319	0.9681	malaria
71	0.9189	0.8848	0.0341	0.9659	typhoid
72	0.9334	0.8928	0.0406	0.9594	typhoid
73	0.7635	0.7423	0.0212	0.9788	malaria
74	0.8903	0.8644	0.0259	0.9741	malaria
75	0.8384	0.8192	0.0192	0.9808	typhoid
76	0.7217	0.6911	0.0306	0.9694	neither
77	0.7979	0.7533	0.0446	0.9554	malaria
78	0.8142	0.7864	0.0278	0.9722	malaria
79	0.9125	0.8881	0.0244	0.9756	typhoid
80	0.7058	0.6953	0.0105	0.9895	neither
**Total**			**1.297**	**38.703**	

DROM = Diagnosis Results of the Orthodox Method, DRPS = Diagnosis Results of the Proposed.

System, $e_{i}$ = error in diagnosis, and (1$-\;e_{i}$) = accuracy of the proposed system.

**Table 6c:** Comparative analysis.

Patient ID	DROM	DRPS	$e_{i}$	(1$-\;e_{i}$)	Disease status
81	0.8201	0.8084	0.0117	0.9883	malaria
82	0.7872	0.7752	0.012	0.988	malaria
83	0.7922	0.7835	0.0087	0.9913	malaria typhoid
84	0.7672	0.7557	0.0115	0.9885	typhoid
85	0.9092	0.8961	0.0131	0.9869	typhoid
86	0.7978	0.7874	0.0104	0.9896	malaria
87	0.8770	0.8661	0.0109	0.9891	typhoid
88	0.8549	0.8472	0.0077	0.9923	malaria
89	0.7406	0.7391	0.0015	0.9985	malaria
90	0.8974	0.8891	0.0083	0.9917	malaria typhoid
91	0.8808	0.8781	0.0027	0.9973	malaria typhoid
92	0.7914	0.7847	0.0067	0.9933	malaria
93	0.8748	0.8531	0.0217	0.9783	typhoid
94	0.7087	0.6859	0.0228	0.9772	neither
95	0.7847	0.7781	0.0066	0.9934	malaria
96	0.8816	0.8791	0.0025	0.9975	typhoid
97	0.8988	0.8869	0.0119	0.9881	typhoid
98	0.8903	0.8731	0.0172	0.9828	malaria
99	0.8767	0.8634	0.0133	0.9867	typhoid
100	0.7775	0.7676	0.0099	0.9901	malaria
101	0.8770	0.8696	0.0074	0.9926	malaria
102	0.8704	0.8583	0.0121	0.9879	typhoid
103	0.8435	0.8247	0.0188	0.9812	malaria typhoid
104	0.7963	0.7819	0.0144	0.9856	malaria
105	0.8764	0.8665	0.0099	0.9901	malaria typhoid
106	0.7327	0.7172	0.0155	0.9845	malaria
107	0.7619	0.7583	0.0036	0.9964	typhoid
108	0.7312	0.7181	0.0131	0.9869	typhoid
109	0.7471	0.7393	0.0078	0.9922	malaria
110	0.8396	0.8175	0.0221	0.9779	malaria
111	0.8138	0.8005	0.0133	0.9867	malaria typhoid
112	0.7819	0.7783	0.0036	0.9964	malaria
113	0.7908	0.7877	0.0031	0.9969	malaria
114	0.9330	0.9275	0.0055	0.9945	typhoid
115	0.8079	0.7819	0.026	0.974	malaria typhoid
116	0.7819	0.7783	0.0036	0.9964	malaria
117	0.8056	0.7883	0.0173	0.9827	malaria
118	0.7755	0.7505	0.025	0.975	typhoid
119	0.7838	0.7701	0.0137	0.9863	malaria
120	0.9010	0.8976	0.0034	0.9966	malaria
**Total**			**0.4503**	**39.5497**	

DROM = Diagnosis Results of the Orthodox Method, DRPS = Diagnosis Results of the Proposed.

System, $e_{i}$ = error in diagnosis, and (1$-\;e_{i}$) = accuracy of the proposed system.

Therefore, the mean accuracy of the proposed diagnosis system and its efficiency is computed as follows:

$$Mean\;Accuracy\;\left(MA\right)=\frac{\sum_{i=1}^{3}\left(1-e_{i}\right)}{n}=\frac{116.4092}{120}=0.970077$$

Efficiency(E)=MA∗100=0.970077∗100=97%

From the evaluation result, it can be concluded that the proposed diagnosis system is most efficient at providing diagnosis for malaria and malaria typhoid at 97% accuracy.

The calculated MA and E values suggested that the proposed system is 97% accurate. In order to show the significant difference in the diagnosis scores between DROM and DRPS, we use the t-test statistics. It is used to test for differences between the two diagnosis methods based on the measured scores. In Table 7, t-test statistics for the two diagnosis methods under comparison was presented. It can be reported in Table 7 that the p-value of 2.24734E-29 means that the averages for DROM and DRPS are significantly different. In order words, the mean values of diagnosis from the orthodox system differ from those of the proposed system.

**Table 7:** t-test statistics.

	DROM	DRPS
Mean	0.81728	0.788273333
Variance	0.004397582	0.004419468
Observations	120	120
Pearson Correlation	0.949619763	
Hypothesized Mean Difference	0	
df	119	
t Stat	15.07592871	
P(T<=t) one-tail	1.12367E-29	
t Critical one-tail	1.657759285	
P(T<=t) two-tail	2.24734E-29	
t Critical two-tail	1.980099876	

In order to conclude that the mean value of the proposed system is statistically the same as that of the orthodox system, equivalence test was investigated. In other words, equivalence test was conducted to investigate if the accuracy of the proposed system is as good as the orthodox system.

Table 8 shows the results of the equivalence test between the orthodox and the proposed diagnosis systems. From the results, it can be concluded that:

• DROM and DRPS scores were strongly and positively correlated (r = 0.947, p < 0.001).
• There was a significant average difference between DROM and DRPS scores ($t_{119}$= 15.091, p < 0.001).
• On average, DROM scores were 0.0299233 points higher than DRPS scores (95% CI [0.0259971, 0.0338496]).

**Table 8:** Equivalence test.

		Mean	N	Std. Deviation	Std. Error Mean
Paired samples statistics	DROM	0.817280	120	0.0663143	0.0060536
	DRPS	0.787357	120	0.0668423	0.0061018
		N	Correlation	Sig.	
Paired correlation	DROM & DRPS	120	0.947	0.000	
		Mean	t	df	Sig. (2-tailed)
Paired differences	DROM-DRPS	0.0299233	15.091	119	0.000
	**Lower**	**Upper**			
95% Confidence Interval (CI) of the difference	0.0259971	0.0338496			

∗Lower = Lower equivalence bound ∗Upper = Upper equivalence bound.

Since the paired mean difference between the orthodox system and the proposed system differ on average by a small score margin of 0.0299233 and probability level for the equivalence test is less than the recommended value of alpha (0.05), the null hypothesis can be accepted and the accuracy of the proposed system can be considered valid.

### 5.1 Conclusion and future work

This paper developed a decision support system for malaria, typhoid fever and malaria typhoid diagnosis using bioinformatics approach. The system is a hybrid of expert system and global alignment with constant penalty. Both malaria and typhoid fever have similar symptoms and are famous for their co-existence in the human body. Hence, the need for an efficient method for detecting these disease conditions.

The architecture of the proposed system takes input diagnosis variables through the browser representing benchmark diagnosis sequences for the three disease conditions, patient signs and symptoms controlled by a health professional and domain expert knowledge respectively. The browser is the interface through which a health professional interact with the system and provide diagnosis results to the outside world. The Knowledge base comprises of the database and rule base. The database stores benchmark diagnosis sequences, patient signs and symptoms and domain expert knowledge while the rule base is made up of a set of IF-THEN rules depicting the benchmark diagnosis variables for each disease conditions of malaria, typhoid fever and malaria typhoid respectively. The matching engine component receives as input the input sequence and applies global alignment technique with constant penalty for the matching between the input sequence and the three benchmark sequences in turns. The global alignment technique with constant penalty applies its pre-defined process to generate optimal alignment and determine the disease condition of the patient through comparing the alignment scores for the three benchmark diagnosis sequences.

We used ANOVA to compare the means of the three independent groups (malaria, typhoid and malaria typhoid) to determine whether there is statistical evidence that the associated values on the diagnosis variables means are significantly different. The ANOVA results indicates that the mean of the values on diagnosis variables is significantly different for at least one of the disease status groups. Similarly, multiple comparisons tests was further used to explicitly tell us which means were different from one another. The multiple comparisons results showed that there is a statistically significant difference in the values on the diagnosis variables to diagnose the disease conditions between the group of malaria and malaria typhoid. Conversely, there were no differences between the groups of malaria and typhoid fever as well as between the groups of typhoid fever and malaria typhoid.

In order to show the significant difference in the diagnosis scores between DROM and DRPS, t-test statistics was used. It is used to test for differences between the two diagnosis methods based on the measured scores. The results of the t-test statistics indicates that the mean values of diagnosis from the orthodox system differ from those of the proposed system. Equivalence test was also conducted to investigate if the accuracy of the proposed system is as good as the orthodox system. The result of the equivalence test validates the accuracy of the proposed system.

Finally, the evaluation of the proposed system showed a high efficiency for the possibility of malaria and malaria typhoid diagnosis. One limitation of the proposed system is related to the age of the sick person. The likelihood that children will not give accurate details of symptoms without a health professional doing physical investigation on the sick children is a clear limitation. In the future, the system will include physical examination category for sick children. Secondly, it is recommended that reinforcement learning be adapted to improve the optimality of the sequence alignment method.

## Declarations

### Author contribution statement

F. E. Ayo: Conceived and designed the experiments; Performed the experiments; Analyzed and interpreted the data; Wrote the paper.

J. B. Awotunde: Performed the experiments.

R. O. Ogundokun: Analyzed and interpreted the data.

S. O. Folorunso, A. O. Adekunle: Contributed reagents, materials, analysis tools or data.

### Funding statement

This research is fully sponsored by Landmark University Centre for Research and Development, Landmark University, Omu-Aran, Kwara State, Nigeria.

### Competing interest statement

The authors declare no conflict of interest.

### Additional information

No additional information is available for this paper.

## References

[bib1] Abatcha M.G., Effarizah M.E., Rusul G. (2019). Antibiotic susceptibility and molecular characterization of Salmonella enterica serovar Paratyphi B isolated from vegetables and processing environment in Malaysia. Int. J. Food Microbiol..

[bib2] Abduah S., Karunamoorthi K. (2016). Malaria and blood transfusion: major issues of blood safety in malaria-endemic countries and strategies for mitigating the risk of Plasmodium parasites. Parasitol. Res..

[bib3] Adehor A.B., Burrell P.R. (2008). An intelligent decision support system for the prompt diagnosis of malaria and typhoid fever in the malaria belt of Africa. IFIP International Conference on Artificial Intelligence in Theory and Practice.

[bib4] Alexopoulos E., Dounias G.D., Vemmos K. (1999). Medical diagnosis of stroke using inductive machine learning. Machine Learning and Applications.

[bib5] Amarathunga A.A.L.C., Ellawala E.P.W.C., Abeysekara G.N., Amalraj C.R.J. (2015). Expert system for diagnosis of skin diseases. Int. J. Sci. Technol. Res..

[bib6] Aminu E.F., Ogbonnia E.O., Shehu I.S. (2016). A predictive symptoms-based system using support vector machines to enhanced classification accuracy of malaria and typhoid coinfection. Int. J. Mathematical Sci. Comput. (IJMSC).

[bib7] Behbahani M., Mohabatkar H., Nosrati M. (2016). Analysis and comparison of lignin peroxidases between fungi and bacteria using three different modes of Chou’s general pseudo amino acid composition. J. Theor. Biol..

[bib8] Boruah I., Kakoty S. (2019). Analytical study of data mining applications in malaria prediction and diagnosis. Int. J. Comput. Sci. Mobile Comput..

[bib9] Bourlas P., Giakoumakis E., Papakonstantinou G. (1999). A knowledge acquisition and management system for ECG diagnosis. Machine Learning and Applications: Machine Learning in Medical Applications.

[bib10] Diniz W., Canduri F. (2017). Bioinformatics: an overview and its applications. Genet. Mol. Res..

[bib11] Djam X.Y., Wajiga G.M., Kimbi Y.H., Blamah N.V. (2011). A fuzzy expert system for the management of malaria. Int. J. Pure Appl. Sci. Technol..

[bib12] Edwards D., Stajich J., Hansen D. (2009). Bioinformatics: tools and applications.

[bib13] Fatumo S.A., Adetiba E., Onaolapo J.O. (2013). Implementation of XpertMalTyph: an expert system for medical diagnosis of the complications of malaria and typhoid. IOSR J. Comput. Eng..

[bib14] Horvitz E.J., Breese J.S., Henrion M. (1988). Decision theory in expert systems and artificial intelligence. Int. J. Approx. Reason..

[bib15] Jan Z., Khan A., Sajjad M., Muhammad K., Rho S., Mehmood I. (2018). A review on automated diagnosis of malaria parasite in microscopic blood smears images. Multimed. Tool. Appl..

[bib16] Lingiardi V., McWilliams N., Bornstein R.F., Gazzillo F., Gordon R.M. (2015). The Psychodynamic Diagnostic Manual Version 2 (PDM-2): assessing patients for improved clinical practice and research. Psychoanal. Psychol..

[bib17] Manickam S., Abidi S.S.R. (1999). Experienced based medical diagnostics system over the World Wide Web (WWW).

[bib18] Manikandan P., Ramyachitra D. (2017). Bacterial foraging optimization–genetic algorithm for multiple sequence alignment with multi-objectives. Sci. Rep..

[bib19] Moses D. (2015). A survey of data mining algorithms used in cardiovascular disease diagnosis from multi-lead ECG data. Kuwait J. Sci..

[bib20] Mutawa A.M., Alzuwawi M.A. (2019). Multilayered rule-based expert system for diagnosing uveitis. Artif. Intell. Med..

[bib21] Needleman S.B., Wunsch C.D. (1970). A general method applicable to the search for similaritiesin the amino acid sequence of two proteins. J. Mol. Biol..

[bib22] Oguntimilehin A., Adetunmbi A.O., Abiola O.B. (2013). A Machine Learning Approach to clinical diagnosis of typhoid fever. Int. J. Comput. Inf. Technol..

[bib23] Pai M., Kalantri S., Dheda K. (2006). New tools and emerging technologies for the diagnosis of tuberculosis: part II. Active tuberculosis and drug resistance. Expert Rev. Mol. Diagn..

[bib24] Poolphol P., Harbach R.E., Sriwichai P., Aupalee K., Sattabongkot J., Kumpitak C., Saeung A. (2017). Natural Plasmodium vivax infections in Anopheles mosquitoes in a malaria endemic area of northeastern Thailand. Parasitol. Res..

[bib25] Qamar F.N., Yousafzai M.T., Khalid M., Kazi A.M., Lohana H., Karim S., Aziz F. (2018). Outbreak investigation of ceftriaxone-resistant Salmonella enterica serotype Typhi and its risk factors among the general population in Hyderabad, Pakistan: a matched case-control study. Lancet Infect. Dis..

[bib26] Rosenberg M.S. (2009). Sequence Alignment: Methods, Models, Concepts, and Strategies.

[bib27] Ruseckaite R. (1999). Computer interactive system for ascertainment of visual perception disorders. Machine Learning and Applications: Machine Learning in Medical Applications.

[bib28] Sajjad M., Khan S., Jan Z., Muhammad K., Moon H., Kwak J.T., Mehmood I. (2016). Leukocytes classification and segmentation in microscopic blood smear: a resource-aware healthcare service in smart cities. IEEE Access.

[bib29] Samuel O.W., Omisore M.O. (2013). Hybrid intelligent system for the diagnosis of typhoid fever. J. Comput. Eng. Inf. Technol..

[bib30] Samuel O.W., Omisore M.O., Ojokoh B.A. (2013). A web based decision support system driven by fuzzy logic for the diagnosis of typhoid fever. Expert Syst. Appl..

[bib31] Shortliffe E.H. (1987). Computer programs to support clinical decision making. J. Am. Med. Assoc..

[bib32] Singla J., Grover D., Bhandari A. (2014). Medical expert systems for diagnosis of various diseases. Int. J. Comput. Appl..

[bib33] Smith T., Waterman M. (1981). Identification of common molecular subsequence. J. Mol. Biol..

[bib34] Uzoka F.M.E., Osuji J., Obot O. (2011). Clinical decision support system (DSS) in the diagnosis of malaria: a case comparison of two soft computing methodologies. Expert Syst. Appl..

[bib35] Wan H.W.I., Fadzilah S. (2006). Artificial Intelligence in Medical Application: an Exploration.

[bib36] Weiss S.M., Kulikowski C.A., Amarel S., Safir A. (1978). A model-based method for computer-aided medical decision-making. Artif. Intell..

[bib37] Yadav G., Pandey G.N. (2015). Development of intelligent decision and prediction system using cyber-enabled NESS technology for oil availability and yield prediction. RIET-IJSET Int. J. Sci. Eng. Technol..

[bib38] Zelic I., Lavrac N., Najdenov P., Rener-Primec Z. (1999). Impact of machine learning of the diagnosis and prognosis of first cerebral paroxysm. Machine Learning and Applications: Machine Learning in Medical Applications.

