# Corrigendum to “A decision support system for multi-target disease diagnosis: A bioinformatics approach”[Heliyon 6 (3), (March 2020) Article e03657]

**DOI:** 10.1016/j.heliyon.2022.e10754

**Published:** 2022-09-27

**Authors:** Femi Emmanuel Ayo, Joseph Bamidele Awotunde, Roseline Oluwaseun Ogundokun, Sakinat Oluwabukonla Folorunso, Adebola Olayinka Adekunle

**Affiliations:** a Department of Physical and Computer Sciences, McPherson University, Seriki Sotayo, Ogun State, Nigeria; b Department of Computer Science, University of Ilorin, Ilorin, Kwara State, Nigeria; c Department of Computer Science, Landmark University, Omu Aran, Kwara State, Nigeria; d Department of Mathematical Sciences, Olabisi Onabanjo University, Ago Iwoye, Ogun State, Nigeria; e Department of Computer Science, Adeyemi College of Education, Ondo State, Nigeria

In the original published version of this article, an error was present in Figure-1. Specifically, the citation for Figure-1 was incorrect. The correct reference for this figure is: “Figure by Deanne Taylor, keynote presentation at the Philadelphia Chapter of the Women in Data Symposium, Philadelphia, April 6, 2019”. The correct citation along with the figure is displayed below. Both the HTML and PDF versions of the article have been updated to correct the error.

**Figure 1:**
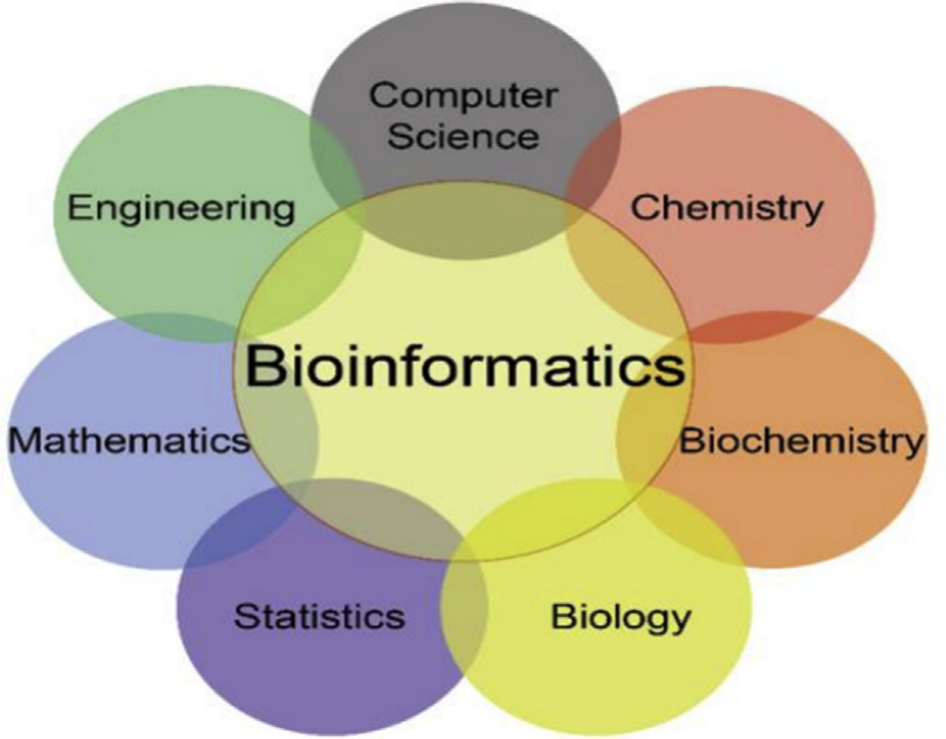
Bioinformatics disciplines (**Source**: Figure by Deanne Taylor, keynote presentation at the Philadelphia Chapter of the Women in Data Symposium, Philadelphia, April 6, 2019).

## Declaration of interests statement

The authors declare no conflict of interest.

