# Corrigendum to “A decision support system for multi-target disease diagnosis: A bioinformatics approach” [Heliyon Volume 6, Issue 3, March 2020, Article e03657]

**DOI:** 10.1016/j.heliyon.2025.e43402

**Published:** 2025-05-22

**Authors:** Femi Emmanuel Ayo, Joseph Bamidele Awotunde, Roseline Oluwaseun Ogundokun, Sakinat Oluwabukonla Folorunso, Adebola Olayinka Adekunle

**Affiliations:** a Department of Computer Sciences, Olabisi Onabanjo University, Ago-Iwoye, Ogun State, Nigeria; b Department of Computer Science, University of Ilorin, Ilorin, Kwara State, Nigeria; c Department of Computer Science, Landmark University, Omu Aran, Kwara State, Nigeria; d Department of Computer Sciences, Olabisi Onabanjo University, Ago-Iwoye, Ogun State, Nigeria; e Department of Computer Science, Adeyemi College of Education, Ondo State, Nigeria

In this article, the ethical approval number for the use of patient database was missing from section 3.2. There is also a minor editing error in the word ‘receive’:

*3.2 Database*

*The database stores patient interaction with the system providing symptoms details. The database is a repository storing the benchmark diagnosis sequences, patient signs and symptoms and domain expert knowledge. It receive input diagnosis variables and the domain expert knowledge through the browser interface. Hence, the authors sought and obtained ethical approval from the Landmark University Research Ethical Board. This ethical approval was given by the Landmark University in collaboration with its medical center.*

The correct version of section 3.2 should be as below:

*3.2 Database*

*The database stores patient interaction with the system providing symptoms details. The database is a repository storing the benchmark diagnosis sequences, patient signs and symptoms and domain expert knowledge. It receive* ***s*** *input diagnosis variables and the domain expert knowledge through the browser interface. Hence, the authors sought and obtained ethical approval from the Landmark University Research Ethical Board. This ethical approval was given by the Landmark University in collaboration with its medical center* ***(LMUIREC/HS/030/2025).***

The authors apologize for the errors.

## Declaration of competing interest

The authors declare no conflict of interest.

